# Composite cardiovascular risk factor control in US adults with diabetes and relation to social determinants of health: The *All of Us* research program

**DOI:** 10.1016/j.ajpc.2025.100939

**Published:** 2025-01-30

**Authors:** Frances Golden, Johnathan Tran, Nathan D. Wong

**Affiliations:** Heart Disease Prevention Program, Division of Cardiology, University of California, C240 Medical Sciences, Irvine, CA 92697, United States

**Keywords:** Diabetes, Cardiovascular disease, Risk factors, Social determinants of health

## Abstract

**Background:**

Data are limited on composite cardiovascular risk factor control in patients with type 2 diabetes mellitus (T2DM). This study aims to identify disparities in cardiovascular risk factor control based on most recent recommendations and relationships to social determinants of health in a large-scale real-world cohort of US adults.

**Methods:**

We analyzed data from 88,416 participants with T2DM in the NIH Precision Medicine Initiative *All of Us* Research Program 2018–2022. We investigated the management of five key cardiovascular risk factors—glycated hemoglobin (HbA1c), LDL cholesterol (LDL-C), body mass index (BMI), blood pressure (BP), and smoking status. Statistical methods included Chi-square tests for categorical comparisons, *t*-tests for mean differences, and multiple logistic regression to assess the impact of demographic and socioeconomic factors on risk factor control.

**Results:**

The study revealed low risk factor control with only 27.7 % of participants achieving recommended levels for three or more risk factors (RFs) and 4.9 % for four or more RFs. Overall, while 81.0% were at target for HbA1c, only 37.9% were at target for BP and 10.4% for LDL-C. Notably, only 1.9 % and 6.9 % were at target for HbA1c, LDL-C, and BP together, based on current and prior recommendations, respectively. Significant disparities were observed across race/ethnicity, sex, and socioeconomic lines with 43.1 % of Asian participants at control for ≥3 RFs compared to 21.1 % of non-Hispanic black participants. In logistic regression analysis, factors such as higher income, higher educational attainment, and health insurance were associated with better RF control, while higher polysocial risk scores linked to poorer control.

**Conclusions:**

Despite some progress in managing individual CVD risk factors in T2DM, overall composite risk factor control remains poor, especially among underrepresented and socioeconomically disadvantaged groups. The findings highlight the necessity for integrated healthcare strategies that address both medical and social needs to improve control of CVD risk factors and outcomes in T2DM.

## Graphical abstract

Central Illustration. Composite Cardiovascular Risk Factor Control in US Adults with Diabetes and Relation to Social Determinants of Health: The All of Us Research Program. Overview of study sample of adults with diabetes, cardiovascular risk factors, social determinants of health, and polysocial risk score and key findings relating to composite cardiovascular risk factor control.

**Figure:**
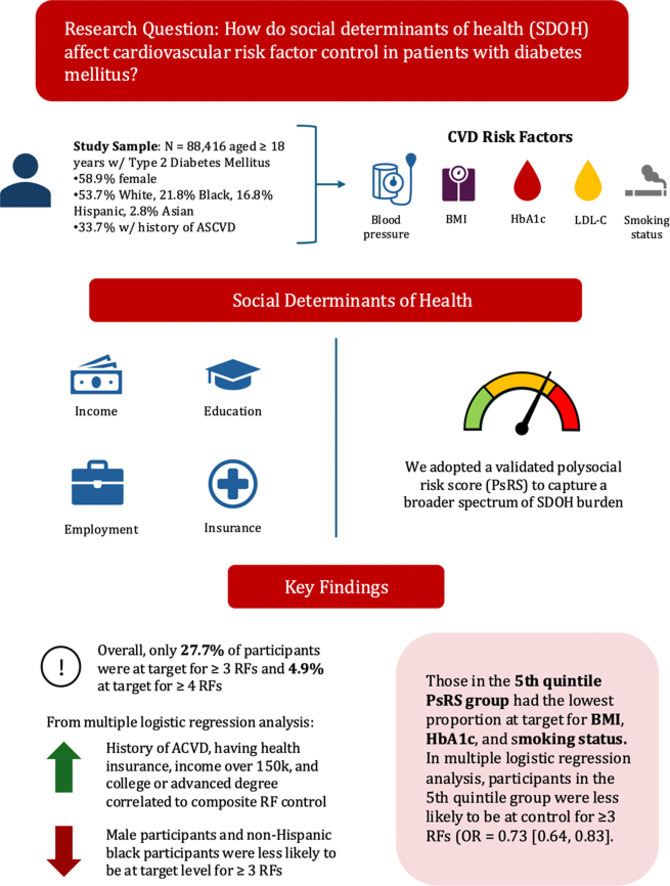

## 1 Introduction

Cardiovascular disease (CVD) is a leading cause of morbidity and mortality in patients with type 2 diabetes mellitus (T2DM), accounting for about 50 % of deaths [1,2]. However, due to lack of current data, T2DM is often underestimated as a risk factor for CVD, underscoring the need for more discussion on this relationship [3]. Healthcare efforts focus on modifiable risks including glycated hemoglobin (HbA1c), blood pressure (BP), and LDL cholesterol (LDL-C), but comprehensive control is low, with prior reports showing only one in five T2DM patients managing these three factors well [[4], [5], [6]]. Research also links social determinants of health (SDOH), such as socioeconomic status, physical environment, food security, and access to healthcare with diabetes outcomes, suggesting that addressing these could significantly lower CVD risk [7,8].

In response to the current disparity in risk factor (RF) management, this study seeks to establish more efficient strategies through understanding CVD risk factor control within the context of singular and overlapping determinants of health. Moreover, there are a lack of data on composite risk factor control based on most recent recommended targets. Utilizing the NIH Precision Medicine Initiative *All of Us* Research Program, we examined the status of CVD risk factor control based on contemporary recommendations in a large sample of US adults with T2DM.

## 2 Methods

The *All of Us* Research Program, launched by the National Institutes of Health's Precision Medicine Initiative, aims to enroll one million participants across the USA. This large-scale biomedical research project focuses on individualized prevention, treatment, and care through an extensive understanding of biological, environmental, and behavioral health determinants. It collects diverse data types such as electronic health records (EHRs), biospecimens (blood, urine, saliva), physical measurements, and wearable device data. As of 2024, the program has recruited over 768,000 participants from >870 sites nationwide, with 80 % from historically underrepresented communities, underlining its commitment to inclusivity [9,10]. Participant consent was obtained either onsite or remotely via the eConsent platform accessible through the program's website or mobile app, with approvals from all participating site institutional review boards [11].

We cleaned and analyzed data using the *All of Us* Researcher Workbench and the built-in application Jupyter Notebook. The data includes electronic health records (EHR), physical measurements (PMs), and surveys completed by participants. EHR data available includes diagnoses, medication history, procedures, and laboratory results. PMs include blood pressure, heart rate, height, weight, and body mass index (BMI). Survey data includes sociodemographic information, overall health, personal and family medical history, lifestyle, substance use, healthcare access and utilization, and other social determinants of health (SDOH) [10]. All participants completed three basic surveys (“The Basics,” “Lifestyle,” “Overall Health”) with the option to complete further surveys on SDOH and COVID-19. Participants were able to opt out of specific questions or additional surveys. The data are pooled from participants of different race/ethnicity, age, sex, and geographic area with the intent to create a dataset that reflects the diversity of the United States [9].

Our study cohort consisted of 88,416 participants aged ≥18 years with type 2 diabetes mellitus (T2DM) mellitus enrolled in the NIH Precision Medicine Initiative All of Us Research Program. We used the data from v7 Curated Data Repository (CDR) released in 2023 containing data collected between 2017 and 2022. Prior to publication of the database, *All of Us* uses the Observational Medical Outcomes Partnership Common Data Model (OMOP CDM) to standardize EHR data from participating sites and ensure participant privacy. T2DM was defined as meeting any of the following criteria: medical history of T2DM (*n* = 49,471), use of diabetes medication or insulin (*n* = 65,540), HbA1c ≥ 6.5 %, non-fasting glucose ≥ 200 mg/dL, or fasting glucose ≥ 126 mg/dL) (*n* = 44,610). Patients with medical history of type 1 diabetes mellitus (*n* = 6347) were excluded from the cohort. Race/ethnicity was collected from survey data and included the categories non-Hispanic White, non-Hispanic Black, Hispanic or Latino, and Asian. We further categorized participants as having a history of atherosclerotic cardiovascular disease (ASCVD) or having no history of ASCVD.

## 3 Definition and measurements

We included data on age, race/ethnicity, sex, ASCVD history, and hypertension along with measurements/laboratory values including BMI, blood pressure, HbA1c, LDL-C, HDL-C, and triglycerides. Survey data included smoking status, health insurance, income, employment status, education level, healthcare access, psychological distress, and food insecurity. History of ASCVD was defined as having a diagnosis of myocardial infarction, coronary artery disease, peripheral artery disease, or cerebrovascular disease, excluding hemorrhagic stroke.

Our primary measures of interest included BMI, blood pressure, LDL-C, HbA1c, and smoking status—factors shown to reduce cardiovascular disease risk when maintained at target levels [12,13]. We defined targets for five key risk factors as: (1) HbA1c < 7 % in those without ASCVD or < 8 % in those with ASCVD, (2) LDL-C < 55 mg/dL in those with ASCVD or < 70 mg/dL in those without ASCVD, (3) blood pressure < 130/80 mm Hg, (4) BMI < 25 kg/m^2^, and (5) smoking status as current, never, or former smoker based on recommendations from the American Diabetes Association (ADA) and/or American Heart Association (AHA) [14,15]. Notably, for BMI the target level was chosen to be < 25 kg/m^2^ based on the cutpoint above where the ADA recommends intervention, either lifestyle or pharmacotherapy.

We adopted a validated polysocial risk score (PsRS) developed by Javed et al. for atherosclerotic disease, based on seven key social determinants: (1) unemployment, (2) inability to pay medical bills, (3) low income, (4) psychological distress, (5) delayed care due to lack of transport, (6) education level less than high school, and (7) food insecurity [16]. This model, refined from 38 potential factors to the most impactful seven, assigns scores from 0 to 20, with a score of 20 indicating most disadvantaged. Previous findings by Javed et al. indicated that individuals in the highest PsRS quintile had a nearly fourfold increase in ASCVD prevalence compared to the lowest quintile [16]. The All of Us survey questions aligned with those used by Javed et al., substituting psychological distress assessment with the Cohen Perceived Stress Scale, where a score of 14 or more signifies distress [17]. A subset of 14,456 participants had complete information for calculating PsRS resulting in a smaller sample size for this analysis.

The R statistical software available on the *All of Us* Research Program Researcher Workbench was utilized for our analysis. We used the Chi-square test of proportions to compare the percent at target level for all five RFs, at target level for the combination of HbA1c, BP, and LDL-C, and at target for 3 and 4 risk factors by history of ASCVD, sex, race/ethnicity, employment, income, education, insurance, and calculated PsRS. We used the Student's *t*-test to compare the mean number of RFs at target across these groups. Multiple logistic regression was used to examine the impact of race/ethnicity, sex, socioeconomic status, insurance, and PsRS, adjusted for age, on the control of three or more RFs.

## 4 Results

A total of 88,416 participants with T2DM were identified of which 29,818 (33.7 %) had a history of ASCVD. Our sample was 58.9 % female with a mean age of 63.8 years and a racial/ethnic breakdown of 53.7 % non-Hispanic White, 21.8 % non-Hispanic Black, 16.8 % Hispanic or Latino, and 1.8 % Asian persons. A majority (95.5 %) had health insurance, with 22.7 % on Medicaid. Socioeconomically, 11.7 % had less than a high school education, 67.8 % were not employed for wages or self-employed, and 17.7 % earned under $10k annually (Table 1).

**Table 1:** Demographic characteristics of participants with T2DM.

Variable	Total (*N* = 88,416)
Age (years) (mean ± SD)	63.8 (14.0)
Sex	
Male	35,407 (41.1 %)
Female	50,639 (58.9 %)
Race/Ethnicity	
Non-Hispanic White	47,480 (53.7 %)
Non-Hispanic Black	19,286 (21.8 %)
Hispanic or Latino	14,872 (16.8 %)
Asian	1623 (1.8 %)
Other race/ethnicity	1515 (1.7 %)
Has health insurance	81,876 (95.5 %)
Has Medicaid	16,568 (22.7 %)
Income	
<10k	12,012 (17.7 %)
10k-25k	12,452 (18.4 %)
25k-50k	13,431 (19.8 %)
50k-75k	8981 (13.2 %)
75k-100k	6436 (9.5 %)
100k-150k	7304 (10.8 %)
Over 150k	7170 (10.6 %)
Education	
Less than high school degree or equivalent	10,010 (11.7 %)
Grade Twelve or GED	18,441 (21.5 %)
Some college	24,468 (28.6 %)
College graduate or advanced degree	32,660 (38.2 %)
Employed for wages	27,534 (67.8 %)
History of ASCVD	29,818 (33.7 %)
History of hypertension	53,740 (62.1 %)
BMI, kg/m²	
< 18.5	725 (0.9 %)
18.5 - 24.9	13,510 (16.1 %)
25 - 29.9	23,666 (28.1 %)
≥ 30	46,234 (55.0 %)
Smoking status	
Never smoked	46,715 (54.6 %)
Former smoker	25,638 (29.9 %)
Current smoker	13,261 (15.5 %)
HbA1c	
< 7 %	47,202 (77.0 %)
7 % -< 8 %	6410 (10.5 %)
>= 8 %	7717 (12.6 %)
Systolic blood pressure (mmHg) (mean ± SD)	130.8 (19.1)
Diastolic blood pressure (mmHg) (mean ± SD)	78.7 (12.0)
LDL-C (mg/dL) (mean ± SD)	101.3 (31.8)
HDL-C (mg/dL) (mean ± SD)	49.9 (15.4)

**Abbreviations:** SD = standard deviation, BMI = body mass index, HbA1c = glycated hemoglobin, LDL-C = low-density lipoprotein cholesterol, HDL-C = high-density lipoprotein cholesterol.

**Fig. 1:**
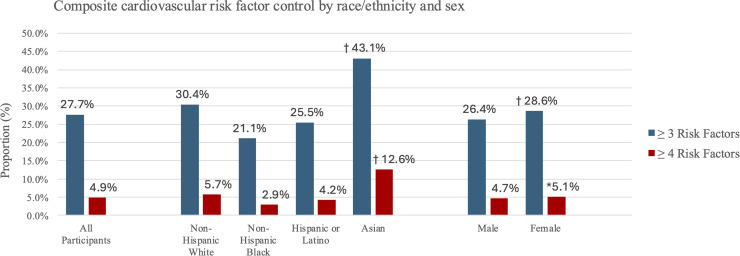
**Composite cardiovascular risk factor control by race/ethnicity and sex.** Risk factors assessed include body-mass index, blood pressure, glycated hemoglobin, low-density lipoprotein cholesterol, and smoking status. P-values represent comparisons between race/ethnicity groups and between males and females. *p < 0.05 †*p* < 0.001.

There were significant differences in percent of participants at target for risk factors by history of ASCVD, sex, and race/ethnicity, illustrated in Table 2. Overall, only 27.7 % of the study sample were at target for ≥ 3 risk factors. Moreover, control of HbA1c, BP, and LDL-C in combination was low with only 1.9 % of the sample at target (6.9 % based on prior recommendations for LDL-C (< 70 mg/dL in those with ASCVD or < 100 mg/dL in those without ASCVD). Participants with a history of ASCVD had a higher percentage (31.9 %) at target for ≥ 3 risk factors than those without ASCVD (25.5 %, *p* < 0.001). Compared to females, males had a lower proportion (*p* < 0.001) at target for BP (34.5 % vs 40.2 %), HbA1c (80.1 % vs 81.7 %), and smoking status (82.3 % vs 86.2 %) but a higher proportion at target for LDL-C (13.6 % vs 8.2 %, *p* < 0.001) and composite control of HbA1c, BP, and LDL-C together (2.4 % vs 1.6 %, *p* < 0.001). Among race/ethnicity, Asian participants had the highest proportion at target for 3 or more RF (43.1 %) and non-Hispanic Black participants had the lowest (21.1 %, *p* < 0.001) as shown in Fig. 1. Asian participants also had the highest proportion at composite control of HbA1c, BP, and LDL-C (3.2 %).

**Table 2:** Cardiovascular risk factor control by history of ASCVD, sex, and race/ethnicity.

		Hx of ASCVD		Sex		Race/Ethnicity				
Proportion (%)	Total (*N* = 88,416)	Yes (*N* = 29,818)	No (*N* = 58,598)	Male (*N* = 35,407)	Female (*N* = 50,639)	Non-Hispanic White (*N* = 47,480)	Non-Hispanic Black (*N* = 19,286)	Hispanic/ Latino (*N* = 14,872)	Asian (*N* = 1623)	Other race/ ethnicity (*N* = 1515)
Individual risk factors										
BMI at target	16.4 %	17.1 %†	16.1 %	16.7 %	16.2 %	17.5 %†	14.9 %	12.2 %	37.0 %	17.5 %
BP at target	37.9 %	37.7 %	37.9 %	34.5 %†	40.2 %	39.5 %†	31.5 %	40.1 %	40.7 %	41.8 %
HbA1c at target	81.0 %	87.4 %†	77.1 %	80.1 %†	81.7 %	86.9 %†	76.2 %	69.1 %	83.5 %	84.4 %
LDL-C at target	10.4 %	8.4 %†	11.7 %	13.6 %†	8.2 %	9.6 %†	11.5 %	11.2 %	11.5 %	10.7 %
Smoking status at target	84.5 %	84.3 %	84.6 %	82.3 %†	86.2 %	88.6 %†	71.2 %	87.6 %	94.6 %	83.3 %
HbA1c, BP, & LDL-C at target	1.9 %	2.0 %	1.9 %	2.4 %†	1.6 %	2.0 %†	1.7 %	1.9 %	3.2 %	1.7 %
≥ 3 risk factors at target	27.7 %	31.9 %†	25.5 %	26.4 %†	28.6 %	30.4 %†	21.1 %	25.5 %	43.1 %	30.7 %
≥ 4 risk factors at target	4.9 %	5.6 %†	4.6 %	4.7 %*	5.1 %	5.7 %†	2.9 %	4.2 %	12.6 %	5.4 %
Risk factors at target (mean ± SD)	1.98 (0.94)	2.10 (0.91) †	1.91 (0.94)	1.94 (0.94) †	2.00 (0.93)	2.06 (0.92) †	1.76 (0.95)	1.93 (0.98)	2.38 (0.98)	2.04 (0.94)

**Abbreviations:** ASCVD = asthersclerotic cardiovascular disease, BMI = body mass index, BP = blood pressure, HbA1c = glycated hemoglobin, LDL-C = low-density lipoprotein cholesterol, SD = standard deviation.

⁎ *p* value < 0.05.

† *p* value < 0.001 compared to no history of ASCVD, female, and across race/ethnicity categories.

Table 3 shows risk factor control by employment, education, income, and insurance. Employed individuals had the highest control rates (*p* < 0.001) for blood pressure (BP) (38.7 %) and smoking status (89.5 %), but not for BMI (14.5 %) and LDL-C (8.5 %). College graduates or those with higher degrees were most often at target for all risk factors, except LDL-C, and significantly more likely to meet ≥ 3 risk factor targets (35.0 %) compared to those with less than a high school diploma (21.4 %, *p* < 0.001). Income correlates with risk factor (RF) control; those earning over $150k had best control of BMI, BP, HbA1c, smoking status, ≥ 3 RFs, and ≥ 4 RFs. Thos earning under $10k were least likely to have BP, HbA1c, smoking status, ≥ 3 RFs, and ≥ 4 RFs controlled. Insured individuals showed significantly better control for ≥ 3 RFs and ≥ 4 RFs (28.2 % and 5.1 %, respectively) than uninsured individuals (18.2 % and 2.6 %, *p* < 0.001). Composite risk factor control by demographic, social determinants of health, and polysocial risk score is summarized in the Central Illustration.

**Table 3:** Cardiovascular risk factor control by employment, education, income, and insurance.

		Individual risk factors at target (%)							
		BMI	BP at target	HbA1c at target	LDL-C at target	Smoking status at target	≥ 3 RFs at target (%)	≥ 4 RF at target (%)	RFs at target (mean ± SD)
Employment	Employed (*N* = 27,534)	14.5 %†	38.7 %†	80.7 %	8.5 %†	89.5 %†	28.1 %†	5.0 %	2.00 (0.92)†
	Unemployed (*N* = 57,861)	17.3 %	37.5 %	81.4 %	11.3 %	82.4 %	27.6 %	4.9 %	1.97 (0.94)
Education	Less than high school degree (*N* = 10,010)	15.5 %†	36.3 %†	67.4 %†	12.6 %†	72.9 %†	21.4 %†	3.3 %†	1.77 (0.95)†
	Grade 12 or GED (*N* = 18,441)	15.0 %	36.0 %	76.2 %	12.1 %	74.9 %	22.8 %	3.4 %	1.81 (0.95)
	Some college (*N* = 24,468)	13.2 %	36.3 %	80.8 %	10.3 %	83.2 %	24.5 %	3.9 %	1.91 (0.91)
	College grad or advanced degree (*N* = 32,660)	19.9 %	40.7 %	88.5 %	9.0 %	94.9 %	35.0 %	7.1 %	2.19 (0.91)
Income	< 10k (*N* = 12,012)	17.3 %†	35.6 %†	73.8 %†	12.8 %†	63.1 %†	20.9 %†	3.0 %†	1.71 (0.98)†
	10k ∼ 25k (*N* = 12,452)	13.8 %	37.1 %	78.3 %	11.6 %	76.8 %	24.2 %	4.0 %	1.87 (0.94)
	25k ∼ 50k (*N* = 13,431)	12.8 %	36.7 %	81.1 %	9.9 %	87.3 %	26.2 %	4.0 %	1.95 (0.91)
	50k ∼ 75k (*N* = 8981)	13.6 %	38.0 %	85.1 %	9.2 %	92.5 %	28.5 %	4.8 %	2.04 (0.88)
	75k ∼ 100k (*N* = 6436)	16.1 %	39.1 %	87.4 %	9.3 %	94.2 %	31.9 %	5.9 %	2.12 (0.89)
	100k ∼ 150k (*N* = 7304)	18.0 %	39.2 %	89.6 %	8.5 %	96.3 %	33.0 %	6.2 %	2.15 (0.88)
	> 150k (*N* = 7170)	23.8 %	41.6 %	92.9 %	8.0 %	97.5 %	38.7 %	9.0 %	2.28 (0.90)
Insurance Status	Insured (*N* = 81,876)	16.4 %	38.0 %†	82.1 %†	10.3 %†	85.3 %†	28.2 %†	5.1 %†	1.99 (0.93)†
	Not insured (*N* = 3840)	16.3 %	35.2 %	60.6 %	12.0 %	70.9 %	18.3 %	2.6 %	1.69 (0.93)
Insurance Type	Medicaid (*N* = 16,568)	15.6 %*	38.1 %	73.8 %†	12.6 %†	69.8 %†	22.6 %†	3.5 %†	1.78 (0.98)†
	Other (*N* = 56,321)	16.6 %	38.0 %	84.4 %	10.0 %	89.8 %	29.5 %	5.4 %	2.05 (0.91)

**Abbreviations:** BMI = body mass index, BP = blood pressure, HbA1c = glycated hemoglobin, LDL-C = low-density lipoprotein cholesterol, RF = risk factor, SD = standard deviation.

⁎ *p* value < 0.05.

† p value < 0.001 compared to unemployed, not insured, “other” insurance type, and across education and income categories.

**Fig. 2:**
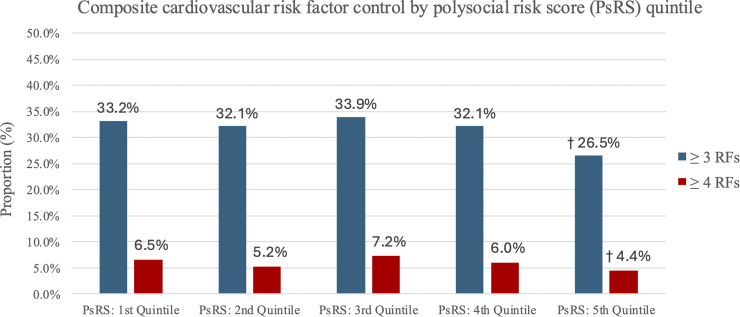
**Composite cardiovascular risk factor control by polysocial risk score (PsRS) quintile**. Risk factors assessed include body-mass index, blood pressure, glycated hemoglobin, low-density lipoprotein cholesterol, and smoking status. PsRS scores range from 0 to 20, with 20 correlating with larger polysocial burden and PsRS: 5th Quintile representing highest PsRS scores. P-values represent comparisons across polysocial risk score quintile groups. *p < 0.05 †*p* < 0.001.

There were significant (*p* < 0.001) differences in control of BMI, HbA1c, and smoking status across quintile of polysocial risk score, with the 5th quintile associated with greatest SDOH burden where the lowest proportion of patients were at target (Table 4). Fig. 2 shows composite cardiovascular risk factor control across risk score quintiles. The 5th quintile group had only 26.5 % at target for ≥ 3 RFs and 4.4 % at target for ≥ 4 RFs compared to 33.2 % (≥ 3 RFs) and 6.5 % (≥ 4 RFs) in the 1st quintile group (*p* < 0.001).

**Table 4:** Cardiovascular risk factor control by quintiles of polysocial risk score (PsRS).

Proportion (%)	Total (*N* = 14,456)	1st Quintile (*N* = 2130)	2nd Quintile (*N* = 1920)	3rd Quintile (*N* = 4317)	4th Quintile (*N* = 3595)	5th Quintile (*N* = 2494)
Individual risk factors						
BMI at target	16.2 %	17.7 %†	15.3 %	19.3 %	15.4 %	11.2 %
Blood pressure at target	39.2 %	39.3 %	40.7 %	37.7 %	40.6 %	38.4 %
HbA1c at target	88.1 %	90.5 %†	86.6 %	91.4 %	87.7 %	81.9 %
LDL-C at target	8.7 %	6.5 %†	7.1 %	9.3 %	9.3 %	9.9 %
Smoking status at target	92.7 %	96.0 %†	92.9 %	96.0 %	91.7 %	85.1 %
≥ 3 risk factors at target	31.8 %	33.2 %†	32.1 %	33.9 %	32.1 %	26.5 %
≥ 4 risk factors at target	6.1 %	6.5 %†	5.2 %	7.2 %	6.0 %	4.4 %
Risk factors at target (mean ± SD)	2.12 (0.90)	2.17 (0.87)†	2.10 (0.90)	2.19 (0.88)	2.11 (0.91)	1.95 (0.92)

**Abbreviations:** BMI = body mass index, HbA1c = glycated hemoglobin, LDL-C = low-density lipoprotein cholesterol, SD = standard deviation.

† *p* value < 0.001 for all factors except blood pressure across quintiles of PsRS.

From multiple logistic regression analyses (Tables 5, 6), male participants (OR = 0.77 [0.74, 0.80]) and non-Hispanic Black participants (OR = 0.81 [0.77, 0.85]) were less likely to be at target level for ≥ 3 RFs. In addition, history of ASCVD (OR = 1.43 [1.37, 1.48]), having health insurance (OR = 1.45 [1.29, 1.41]), income of over 150k (OR = 1.66 [1.52, 1.80]), and college or advanced degree (OR = 1.58 [1.45, 1.71]) were associated with a greater likelihood of composite RF control. With a reference group of the 1st quintile, participants in the 5th quintile for PsRS scores were least likely to be at target for ≥ 3 RFs (OR = 0.73 [0.64, 0.83]). In contrast, when examining the odds of being at target for HbA1c, LDL-C, and BP together, non-Hispanic Black participants had highest odds among race/ethnic groups (OR = 1.20 [1.01, 1.44]), while males were less likely to be at target level (OR = 0.68 [0.60, 0.77]) as were unemployed participants (OR = 0.72 [0.61, 0.84]).

**Table 5:** Multiple logistic regression of indicators for ≥ 3 risk factors at target.

Variable	Odds Ratio [95 % CI] (*N* = 64,105)
Age	1.00 [1.00, 1.00]
Sex: male	0.77 [0.74, 0.80]
Non-Hispanic Black	0.81 [0.77, 0.85]
Hispanic or Latino	1.02 [0.95, 1.08]
Asian	1.58 [1.40, 1.79]
Other race/ethnicity	1.32 [0.99, 1.29]
History of ASCVD	1.43 [1.37, 1.48]
Has health insurance	1.45 [1.29, 1.63]
Unemployed	1.09 [1.04, 1.14]
Income: 10k-25k	1.07 [1.01, 1.42]
Income: 25k- 50k	1.12 [1.04, 1.20]
Income: 50k - 75k	1.15 [1.07, 1.24]
Income: 75k - 100k	1.29 [1.19, 1.41]
Income: 100k - 150k	1.33[1.23, 1.44]
Income: > 150k	1.66 [1.52, 1.80]
Education: Grade Twelve/GED	1.07 [0.99, 1.16]
Education: Some college	1.09 [1.00, 1.18]
Education: College grad or advanced degree	1.58 [1.45, 1.71]

**Reference groups**: Sex – female, race/ethnicity – Non-Hispanic White, history of ASCVD – no history of ASCVD, health insurance – uninsured, income – <10k, employment – employed for wages, education – less than high school degree or equivalent.

**Table 6:** Multiple logistic regression of indicators using polysocial risk score (PsRS) for ≥ 3 risk factors at target.

Variable	Odds Ratio [95 % CI] (*N* = 14,276)
Age	1.00 [1.00, 1.01]
Sex: male	0.76 [0.71, 0.83]
Non-Hispanic Black	0.71 [0.62, 0.81]
Hispanic or Latino	0.91 [0.77, 1.06]
Asian	1.98 [1.51, 2.60]
Other race/ethnicity	0.96 [0.71, 1.28
History of ASCVD	1.46 [1.35, 1.58]
PsRS: 2nd Quintile	0.97 [0.85, 1.11]
PsRS: 3rd Quintile	0.96 [0.86, 1.08]
PsRS: 4th Quintile	0.92 [0.82, 1.03]
PsRS: 5th Quintile	0.73 [0.64, 0.83]

**Reference groups**: Sex – female, race/ethnicity – Non-Hispanic White, history of ASCVD – no history of ASCVD, PSRS – 1st Quintile.

## 5 Discussion

In a real-world cohort of US adults with T2DM from the NIH Precision Medicine Initiative All of Us research program which was highly diverse (nearly 60 % female and over 45 % non-White), we demonstrate continuing suboptimal control of multiple cardiovascular risk factors, with important disparities according to key demographic and SDOH factors. Despite modest control of individual risk factors, overall management remains poor, with less than a third having 3 or more risk factors and only 5 % with ≥4 risk factors controlled to recommended levels, respectively. Control of three key risk factors (HbA1c, BP, and LDL-C) is exceedingly low at 1.9 % overall. Those who are male, Asian, or with prior history of ASCVD had significantly better individual and composite risk factor control. Examination of specific social determinants of health including employment, education, income, as well as a validated polysocial risk score (PsRS) indicated a connection between lower socioeconomic burden (high income, higher degree of education, insurance status, lower PsRS) with better composite risk factor control.

With cardiovascular disease (CVD) as a leading cause of mortality and morbidity in patients with T2DM, focus on modifiable cardiovascular disease risk factors has been a pillar of diabetes management. The importance of control of multiple CVD risk factors on CVD outcomes has been previously demonstrated in other cohort studies. Our prior analysis of the Multi-Ethnic Study of Atherosclerosis (MESA), and Jackson Heart Study (JHS), and Atherosclerosis Risk in Communities (ARIC) study demonstrated control of BP, LDL-C, and HbA1c was linked to respective decreases in CVD risk of 17 %, 33 %, and 37 % with 60 % or more lower risks for incident CHD and CVD events when all 3 are controlled [12]. In the BARI 2D trial of 2265 patients with T2DM, Bittner et al. looked at composite CVD risk factor control of 6 key risk factors and the related this to the risk of death finding that patients with 0 to 2 risk factors in control during follow-up had a risk of death 2-fold higher than those with all 6 CVD risk factors of interest at target level [18]. In a 2018 study using the Swedish National Diabetes Register, Rashwani et al. found that patients with diabetes who had 5 modifiable CVD risk factors in control were at similar risk of risk of death, stroke, or myocardial infarction compared to the general population [19].

Despite the focus on modifying risk factors in diabetes management, our study indicates adequate control is poor across with HbA1c and smoking status best controlled overall (81.0 % and 84.5 %, respectively) and BMI and LDL-C least controlled (16.4 % and 10.4 %, respectively). This study builds on findings from our prior report on patients with DM from the National Health and Nutrition Examination Surveys 2013–2016 [6]. In the NHANES cohort, 17.3 % of participants met target levels for HbA1c, BP, and LDL-C. In the All of Us cohort, only 1.9 % achieved target levels for HbA1c, BP, and LDL-C, significantly lower than in NHANES. In both studies, patients with ASCVD history had better HbA1c control but worse LDL-C control compared to those without ASCVD. The discrepancy may reflect the stricter 2023 American Diabetes Association guidelines for LDL-C, which lowered targets from 70 mg/dL and 100 mg/dL in T2DM patients with and without ASCVD to 55 mg/dL and 70 mg/dL, respectively [4]. Notably, our cohort had 81.0 % at target for HbA1c compared to 55.8 % in the NHANES cohort, which may reflect wider availability and use of glucose lowering drugs in the past decade [20,21].

Significant disparities in risk factor control by race/ethnicity and sex exist in our cohort. Asian participants exhibited equal or superior control of all five risk factors compared to non-Hispanic Black participants. Females were more likely to have composite risk factor control. Research highlights an association between race/ethnicity and sex with the prevalence of both T2DM and CVD. Minority groups were disproportionately affected, with non-Hispanic Black persons experiencing nearly double the prevalence of diabetes compared to non-Hispanic White persons [7,22]. Similarly, the prevalence of CVD is higher in non-Hispanic Black persons (46.8 %) compared to non-Hispanic white (36.4 %), Hispanic (31.15 %), and non-Hispanic Asian (29 %) persons [22]. The MESA study found that Black participants had a 34 % higher mortality hazard and Chinese participants a 21 % lower mortality hazard compared to White participants [23]. Racial/ethnic gaps in use of pharmacotherapies targeting modifiable RFs may play a role in these disparate outcomes [24,25]. Addressing modifiable risk factors in these patients may help close the racial and ethnic gaps that exist in CVD prevalence and mortality among patients with diabetes.

Beyond race/ethnicity and sex, our investigation of SDOH found that higher socioeconomic burden is significantly linked to worse composite risk factor control. Those that were insured, had more years of education, and higher income had better cardiovascular risk factor control. Prior research has linked lower socioeconomic status (SES) to increased risks and worse outcomes for both cardiovascular disease (CVD) and type 2 diabetes mellitus (T2DM). A systematic review and meta-analysis by Agardh et al. showed that people with low SES have a higher risk of T2DM, with relative risks of 1.41 for education level, 1.31 for occupation, and 1.40 for income. This trend holds true globally, regardless of a country's income level [26]. An analysis of the National Health Interview Survey by Saydah et al. found that participants with less than a high school education or a family income below the poverty level had double the mortality rate from diabetes compared to those with higher SES [27]. Furthermore, these socioeconomic disparities extend to cardiovascular outcomes, indicating that the impact of SES on health goes beyond diabetes [28]. Acquah et al. also found that greater social disadvantages were linked to higher prevalence of key cardiovascular risk factors, such as hypertension and diabetes, highlighting the significant impact of SES on overall health [29].

Recognizing that the impact of SDOH are not based on solitary factors, but rather a diverse array of interlinking risk factors that affect health, we utilized a validated polysocial risk score (PsRS) that aggregates key determinants for CVD. SDOH have been omitted in traditional cardiovascular risk assessment scores, but when added to these scores improves risk assessment and reduces bias [30,31]. In our cohort, participants in the 5th quintile of the PsRS, indicative of greatest social disadvantage, showed poorer control of BMI, BP, HbA1c, and smoking status compared to those in the 1st quintile. In a multiple logistic regression of indicators for ≥ 3 risk factors at target adjusted for age and race/ethnicity, the 5th quintile had an odds ratio of 0.73 [0.64, 0.83], illustrating the significant impact of compounded social disadvantages on health.

The translational implications of these findings emphasize the need for diabetes and cardiovascular medicine to go beyond traditional interventions to include social determinants of health. The strong association between income, education, and insurance status with composite cardiovascular risk factor control in this study indicate a need to address upstream determinants of health. Clinically, incorporation of validated assessments of social determinants of health into routine preventative care could help identify patients at higher risk for poor cardiovascular outcomes. These high-risk groups may require a more targeted approach from multidisciplinary care teams and community-based programs to promote lifestyle changes, improve medication adherence, and address barriers to care.

Our study has several strengths and limitations. The *All of Us* Research Program dataset is a large, diverse patient population that includes minority groups often overlooked in research. Our sample included nearly 60 % female and nearly 50 % non-White participants, as well as significant proportions of lower income and less well educated individuals. Additionally, we align our analysis with the 2023 updated Standards of Care in Diabetes, incorporating the new LDL-C targets. However, our cross-sectional design in the present study did not allow for the analysis of disease progression or incident CVD or other outcomes. Moreover, while *All of Us* captures a comprehensive sample of US adults, as a research study, it is still subject to participation baises that may limit its generalizability to the entire US population. The findings from our study are most generalizable to diverse populations that mirror the demographic composition of the *All of Us* Research Program cohort, which intentionally oversamples groups underrepresented in biomedical research.

In summary, our study of a diverse U.S. cohort with diabetes demonstrates that, despite heightened awareness and management efforts, control of cardiovascular risk factors remains inadequate, influenced by disparities across sex, race/ethnicity, and ASCVD history. Crucially, poorer control is associated with social determinants such as low income, less education attainment, and being uninsured. This underscores the need to better understand the social obstacles preventing effective management of diabetes. Addressing these barriers through targeted individual and community interventions could significantly improve cardiovascular and other health outcomes for this expanding patient population.

## Disclosures

Dr. Wong has received research funding through his institution from Novo Nordisk, Lilly, Amgen, Novartis, and Regeneron and is a consultant/advisor for Amgen, Novartis, and Heart Lung.

## Funding

No funding was received for this project or preparation of this manuscript.

## Author agreement

Frances Golden, Johnathan Tran and Nathan D. Wong have agreed to the submission of this MS.

## CRediT authorship contribution statement

**Frances Golden:** Writing – review & editing, Writing – original draft, Methodology, Formal analysis. **Johnathan Tran:** Methodology, Formal analysis. **Nathan D. Wong:** Writing – review & editing, Supervision, Project administration, Methodology, Data curation, Conceptualization.

## Declaration of competing interest

Dr. Wong reports research funding through his institution from Amgen, Novo Nordisk, Novartis, and Regeneron separate from this project and is a consultant/advisory board member for Amgen, Novartis, Ionis and HeartLung. The other authors declare there are no conflicts of interested related to the content of this manscript.
